# Stable and Functionally Diverse Versatile Peroxidases Designed Directly from Sequences

**DOI:** 10.1021/jacs.1c12433

**Published:** 2022-02-18

**Authors:** Shiran Barber-Zucker, Vladimir Mindel, Eva Garcia-Ruiz, Jonathan J. Weinstein, Miguel Alcalde, Sarel J. Fleishman

**Affiliations:** † Department of Biomolecular Sciences, Weizmann Institute of Science, Rehovot 7600001, Israel; ‡ Department of Biocatalysis, Institute of Catalysis, CSIC, Cantoblanco, Madrid 28094, Spain

## Abstract

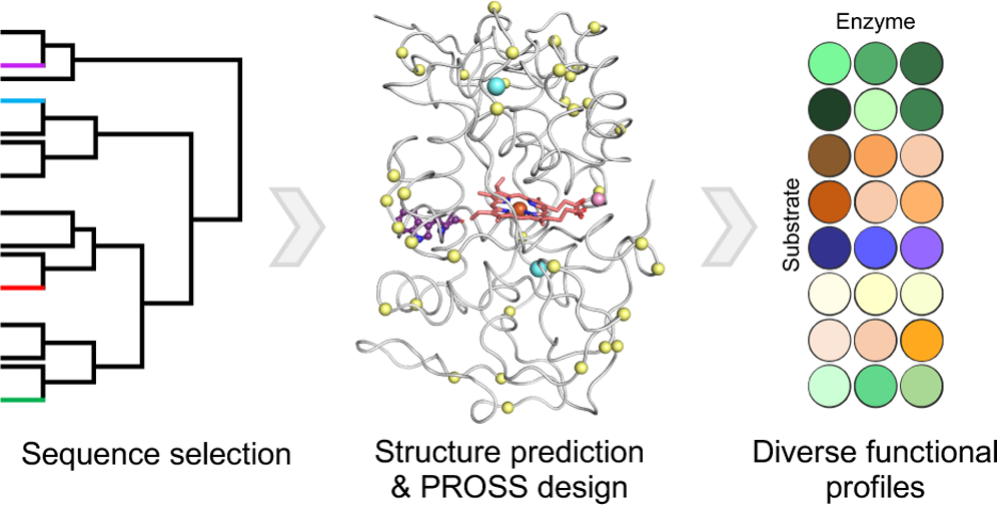

White-rot fungi secrete a repertoire of high-redox potential oxidoreductases to efficiently decompose lignin. Of these enzymes, versatile peroxidases (VPs) are the most promiscuous biocatalysts. VPs are attractive enzymes for research and industrial use but their recombinant production is extremely challenging. To date, only a single VP has been structurally characterized and optimized for recombinant functional expression, stability, and activity. Computational enzyme optimization methods can be applied to many enzymes in parallel but they require accurate structures. Here, we demonstrate that model structures computed by deep-learning-based *ab initio* structure prediction methods are reliable starting points for one-shot PROSS stability-design calculations. Four designed VPs encoding as many as 43 mutations relative to the wildtype enzymes are functionally expressed in yeast, whereas their wildtype parents are not. Three of these designs exhibit substantial and useful diversity in their reactivity profiles and tolerance to environmental conditions. The reliability of the new generation of structure predictors and design methods increases the scale and scope of computational enzyme optimization, enabling efficient discovery and exploitation of the functional diversity in natural enzyme families directly from genomic databases.

## Introduction

The need for developing economical and environmentally friendly energy sources is undeniable. Efficient conversion of biomass, particularly lignocellulose, into biofuels is a promising route for sustainable and renewable energy production.^1^ The amorphous and highly cross-linked structure of lignin, however, obstructs the accessibility of chemicals and enzymes to cellulose and impedes their conversion into biofuels and other high-value chemicals.^2−4^ Furthermore, lignin itself comprises potentially valuable chemicals that could be valorized. Since chemical depolymerization of lignin is still not economically viable or environmentally benign,^2,5^ biodegradation is an attractive route for utilization of wood biomass.

The most efficient natural system for lignin depolymerization is observed in white-rot basidiomycetes. These fungi secrete a repertoire of high-redox potential oxidoreductases (laccases and peroxidases) that degrade lignin synergistically.^6−8^ Of these, versatile peroxidases (VPs; EC 1.11.1.16) are of particular interest for biotechnological use due to their broad substrate scope ranging from low- to high-redox potential substrates. VPs reduce hydrogen peroxide by oxidizing a wide range of substances, including phenolic and nonphenolic compounds, pesticides, high-redox potential dyes, polycyclic aromatic hydrocarbons, and lignin.^9^ Some fungal species secrete several VP paralogs, suggesting that VPs may act synergistically.^10,11^ Nevertheless, VPs are especially challenging for heterologous production, limiting their use in research, let alone as an enzyme repertoire or in industrial applications.

One reason why VPs are functionally promiscuous is that they comprise three distinct active sites for substrate oxidation: a site for the oxidation of Mn^2+^ to Mn^3+^, which acts as a diffusible mediator, a low-redox potential heme-dependent binding pocket, and a high-redox potential surface-reactive tryptophan radical, which connects to the heme through a long-range electron-transfer pathway.^9^ Additionally, they comprise two structural calcium ions, multiple glycosylations, and several disulfide bonds, thereby complicating their expression in heterologous hosts. Thus, to date, only a VP from *Pleurotus eryngii* (VPL) has been fully characterized biochemically and structurally.^12^ Several directed evolution campaigns successfully adjusted VPL to various industrial requirements: functional expression in the yeast *Saccharomyces cerevisiae*, thermostability,^13^ stability and activity in neutral and alkaline pH,^14^ and stability and activity under high concentrations of H_2_O_2_,^15^ which serves as the terminal electron acceptor in VPs and is also a strong inhibitor. In each such campaign, 5000–15 000 clones generated by random mutagenesis and diverse DNA recombination methods were screened to reach the desirable trait.^13−15^ Although successful, the high labor intensity makes directed evolution an impractical approach for optimizing multiple natural starting points. The ability of ancestral sequence reconstruction to optimize VPs is also limited as it can generate only one or few enzymes and therefore cannot expose multiple functional profiles encoded among natural homologues.^11,16^ Thus, computational design methodologies may provide a useful alternative.^17^ The PROSS structure-based algorithm combines phylogenetic sequence information with Rosetta atomistic design to incorporate stabilizing mutations and optimize the native-state energy.^18,19^ PROSS has addressed protein expression and stability problems in challenging proteins by an experimental screening of only a few designs (typically ≤5). In a number of cases, proteins that could not be functionally expressed in microbial systems could be expressed robustly after one-shot design calculations.^18−20^ PROSS is sensitive to atomic details, however, and except in a handful of cases,^21,22^ has not been applied to structural models.

**Figure 1:**
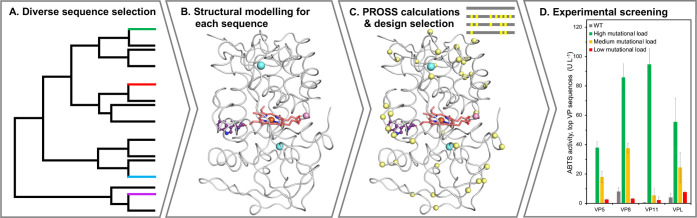
Key steps in the design of a diverse set of VPs. (A) VP sequences were collected from different databases, a phylogenetic tree was computed and 12 representative sequences were selected. (B) Selected sequences were modeled by trRosetta.^25,26^ For visualization, heme (red), manganese (pink), and calcium ions (blue) were superimposed from the VPL structure (PDB entry: 3FJW). The surface-reactive tryptophan is presented in purple balls-and-sticks. (C) PROSS stability-design calculations^18,19^ suggested dozens of mutations (yellow spheres). For each sequence, three designs with different mutational loads were selected for further experimental examination. (D) In an activity screen of proteins heterologously produced in yeast, the wildtype proteins show negligible functional expression, while the designs with the highest mutational load are highly active on the peroxidase substrate ABTS.

Recently, a new generation of deep-learning-based *ab initio* structure prediction methods has been developed, with the most recent ones reaching the accuracy of crystal structures.^23,24^ Here, we introduce a general pipeline for increasing the functional expression yields of proteins for which no experimental structure data are available using these structure predictors. The structural and biochemical complexities of VPs make them especially challenging as a proof-of-concept for this pipeline. First, we selected 11 diverse VP sequences, modeled them using the trRosetta structure prediction algorithm made available in December 2020,^25,26^ and ran PROSS^18,19^ stability-design calculations on each as well as on VPL (in total 12 natural VPs; Figure 1). After screening only 36 designs (three for each wildtype VP), we isolated three efficient, highly stable VPs that can be functionally expressed in yeast, whereas their wildtype progenitors could not be recombinantly expressed efficiently. Our approach exploits nature’s diversity to generate a VP repertoire that can be used to decompose lignin and diverse pollutants. Furthermore, the combination of the structural and experimental data we obtained expands our understanding of the VP structure–function relationship.

## Results

### Computational Design for Functional Expression Directly from Sequences

VP sequences were extracted from public databases, phylogenetically classified, and 11 sequences that exhibited 51–81% identity to one another were selected for modeling using trRosetta (Figure 1A,B and Tables S1 and S2; see Methods, Supporting Information for more details).^25,26^ The top model in each case was then subjected to PROSS stability design (Figure 1C).^18,19^ VPL was also designed by PROSS based on its crystallographic structure (PDB entry: 3FJW). The new structure prediction methods do not model ligands and ions. We visually compared the models with the VPL experimental structure finding that the models retained the intricate arrangement of amino acids in the heme-binding pocket and the ion-binding sites (Figure S1). This observation encouraged us that the trRosetta models could be used as reliable starting points for the design of enzymes as complex as the VPs. Based on the VPL crystallographic structure,^27^ we inferred which positions in each model comprised the catalytic and ion–ligand sites and disabled design in these positions. We also restricted design in positions where model uncertainty was high and in adjacent positions (see Methods, Supporting Information). Finally, we selected the wildtype sequence and three PROSS designs with different mutational loads for experimental characterization (∼10 mutations in the most conservative design and up to 49 in the most permissive one, see Table S1 and several designed sequences in Figure S2). VPs comprise ∼340 amino acids; thus, roughly 12% of the protein template was mutated in the most permissive designs. The DNA encoding each protein was codon-optimized for yeast expression, ordered as synthetic gene fragments that were incorporated into the pJRoC30 plasmid downstream of the *S. cerevisiae* α factor prepro-leader and transformed into yeast cells.

The approach described above yielded four diverse and functionally expressed VPs (Figure 1D): VPL (from *P. eryngii*), two paralogs from *Pleurotus ostreatus* (VP5 and VP11), and a VP from *Ganoderma* sp. 10597_SS1 (VP8) (Table S3). For these VPs, the wildtype progenitors demonstrate poor functional expression while the designs efficiently oxidize the peroxidase substrate 2,2′-azino-bis (3-ethylbenzothiazoline-6-sulfonic acid) (ABTS). For instance, the wildtype enzymes, VP5 and VP11, exhibit no detectable functional expression, and for VP8 and VPL, the best designs exhibit 11-fold and 14-fold increased activity compared to their respective wildtype enzymes (Figure 1D). For all four VPs, the most active design exhibits the highest mutational load (38–43 mutations, >10% of the sequence, Figure S2). The designs are therefore denoted as 5H, 8H, 11H, and VPLH to designate their high mutational load. The designed mutations exhibit improved core packing, introduce new hydrogen-bond networks, and rigidify loops (Figure S3). Some of these mutations are radical (for example, an Ile → Phe mutation in the protein core; Figure S3A), and in many cases, proximal mutations form multiple new contacts (Figure S3B,C), demanding atomic accuracy in the starting models.

### Designed VPs Are Highly Stable

Since the wildtype VPs showed negligible functional expression, all further biochemical analyses were done on 5H, 8H, and 11H relative to the VPL variants R4 and 2-1B (previously evolved for expression and thermostability, respectively).^13^ Although VPLH also demonstrated significantly enhanced functional expression compared to its wildtype protein (Figure 1D), it did not show substantial improvement relative to R4 and 2-1B and was not pursued further.

**Figure 2:**
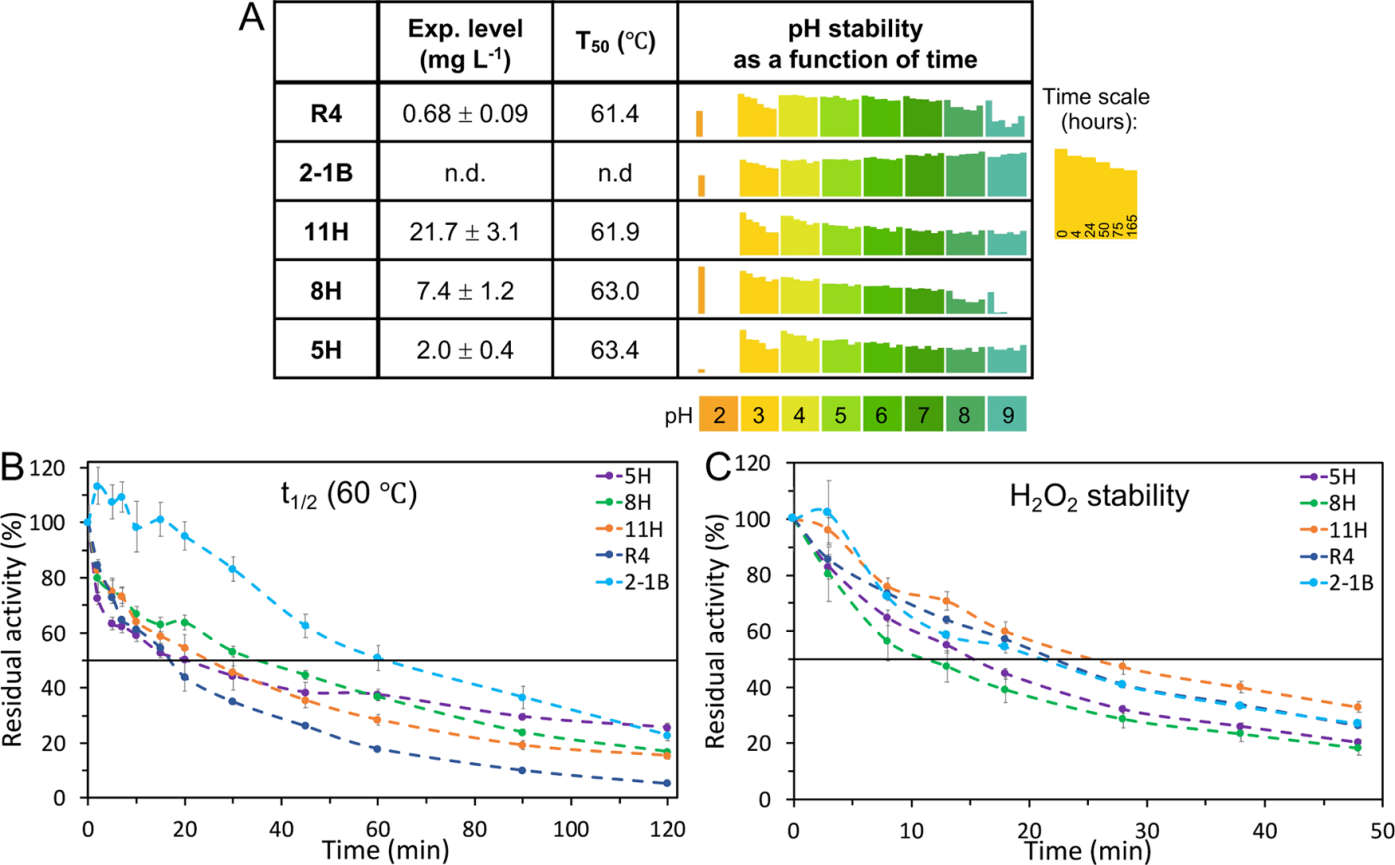
Functional expression levels, thermal, pH, and H_2_O_2_ stability in VP designs. (A) Expression levels were calculated using the initial activity against ABTS in the supernatant immediately after growth and the ABTS kinetic values (see Methods, Supporting Information). Apparent *T*_50_ values were calculated based on the residual activity of enzymes incubated at different temperatures ranging from 25 to 80 °C (see Figure S4A); n.d.: not determined. pH stability was assessed by incubation at pH values ranging from 2 to 9 and measuring the residual activity at times 0, 4, 24, 50, 75, and 165 h, compared to the activity at pH = 3 at time zero (see Figure S5 for complete data). (B) Kinetic thermostability (*t*_1/2_) profiles were determined by incubating VP supernatants at 60 °C and measuring their residual activity at times 0–120 min, compared to the initial activity. (C) H_2_O_2_ stability (*t*_1/2_) profiles were determined by incubating designs in 750 μM H_2_O_2_ (molar ratio of 1:3000) and measuring their residual activity at times 0–48 min, compared to the initial activity. All of the results are expressed as the mean ± S.D. of three independent experiments.

The designed VPs exhibit higher thermal stability compared to R4 (Figures 2A,B and S4). While the temperature at which the enzyme loses half of its maximal activity after 15 min of incubation (*T*_50_) is similar to R4 across all designs, at elevated temperatures, 5H does not lose its activity completely (Figure S4A). In shorter incubations, 5H shows much-enhanced activity at 45–60 °C compared to the activity at room temperature, similar to 2-1B, and the highest residual activity at 65–80 °C (Figure S4B). This trend is consistent with the observed kinetic stability at 60–65 °C (*t*_1/2_, the time at which the protein loses half of its activity after incubation at a specific temperature; Figures 2B and S4C): 5H maintains stable residual activity even after 2 h, comparable to 2-1B, which is evolved specifically to withstand high temperatures. Long-term heat resistance, as observed for these designs, is an important advantage when using enzymes in industrial processes.

In nature, lignin is decomposed under acidic conditions,^11^ and VP activity is strongly acid-dependent.^14,28,29^ Particularly, the formation of the tryptophan radical at the high-oxidation site is facilitated at low pH,^11^ and a high oxidizing power depends on the stability and activity at pH 2–3.^14^ 8H is stable under acidic to neutral pH values and is the most active VP variant after incubation in a highly acidic pH (pH = 2; Figures 2A and S5). VPs may also be useful in alkaline conditions, for instance, for biomedical purposes and for paper and textile processing.^8^ Remarkably, designs 5H and 11H maintain their initial activity levels even after 1 week of incubation at pH 9 (Figures 2A and S5A).

Hydrogen peroxide is the terminal electron acceptor in VPs but, at high concentrations, it also deactivates enzymes.^30^ To assess the stability at high hydrogen peroxide concentrations, VPs were incubated with H_2_O_2_ at a 1:3000 molar ratio (Figure 2C). Of all VP variants, 11H exhibits the highest stability to hydrogen peroxide.

Lastly, we estimated the expression levels of all VPs based on initial rate measurements in the yeast broth derived from screening experiments and the kinetic constant data (see Table S4, Methods, Supporting Information, and below). Under these specific conditions, which were not optimized for high protein expression, the three VP designs show much greater functional expression levels than R4, which is the highest functionally expressed VPL variant^13^ (Figure 2A). Further gains in functional expression can be made by optimizing the experimental conditions or expression strain, as was previously noted in the R4 directed evolution campaign.^13^ We concluded that the designs were stable and exhibited remarkable differences in their tolerance of environmental conditions.

### Designs Exhibit Diverse Functional Profiles

**Figure 3:**
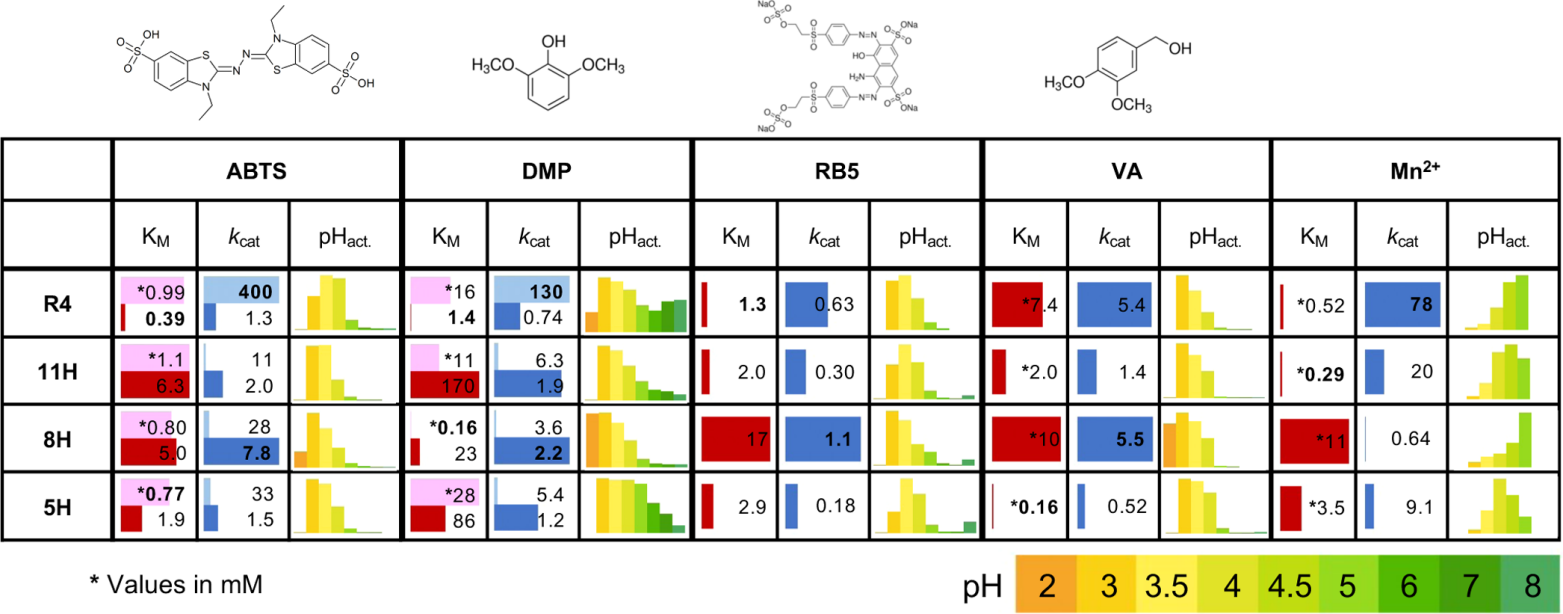
High functional diversity among VP designs. For each VP (5H, 8H, 11H, and R4) and substrate (ABTS, DMP, RB5, VA, and Mn^2+^), two kinetic parameters, *K*_M_ and *k*_cat_, are shown (in μM and s^–1^, respectively; *K*_M_ values given in mM are marked with asterisks); bars are normalized to the larger value in each substrate (worst *K*_M_ and best *k*_cat_). For ABTS and DMP, light bars refer to the kinetic parameters of the low-efficiency site and the dark bars to the high-efficiency site; kinetic values are normalized separately for each (Table S4 shows all kinetic parameters). The best affinity (lowest *K*_M_) and turnover number (highest *k*_cat_) for each substrate are highlighted in bold. pH-dependent activity profiles are shown for each enzyme–substrate pair, and the bars are normalized to the activity at optimal pH for each such pair (Figure S6 shows full activity plots).

We next tested the activity profiles of the top-three VP designs with a range of peroxidase substrates using R4 as a reference (Figures 3 and S6 and Table S4). VPs exhibit a very broad substrate scope. Accordingly, we chose five substrates that represent different redox potentials and chemical structures: ABTS and 2,6-dimethoxyphenol (DMP; low-redox potential substrates), veratryl alcohol (VA; a high-redox potential substrate), reactive black 5 (RB5; a high-redox potential dye), and Mn^2+^. DMP, VA, and Mn^2+^ are likely to be native substrates of white-rot VPs since VPs functionalize them to serve as mediators for lignin oxidation. VA and RB5 are exclusively oxidized by the high-redox potential surface-active tryptophan, while DMP and ABTS are oxidized both in the heme pocket (low-) or by the tryptophan radical (high-efficiency site).^11,12,27,31−34^ Furthermore, Mn^2+^ is oxidized in a distinct active site. Thus, the five substrates probe the reactivity and selectivity of each of the three VP active sites. Remarkably, despite the structural complexity, the lack of cofactors in the model structures, and the very large number of mutations in each design, the designs oxidize all of the substrates (Figure 3). Thus, the combination of *ab initio* modeling and PROSS design is effective even in complex, multifunctional enzymes such as VPs.

The four VPs show the expected preference for acidic conditions (Figures 3 and S6). Nevertheless, pH preferences vary dramatically among the designs. For instance, 8H exhibits a strong preference for a low pH (2–3) in DMP and VA oxidation, whereas 5H is nonreactive at low pH and is reactive at a relatively high pH where 8H is inactive. Furthermore, whereas R4 exhibits relatively broad pH reactivity for DMP, it is more restricted in the pH scope on the other substrates relative to the designs. Manganese oxidation as a function of pH shows unexpected trends. Typically, VPs exhibit a pH optimum of 5 for oxidizing Mn^2+^ (as in R4).^11,13,27,35^ 8H also exhibits a pH optimum of 5, even though this design exhibits a more acidic pH optimum for the other substrates. By contrast, 5H and 11H have a pH optimum at 4 and 4.5, respectively, closer to the native conditions in which white-rot fungi operate (pH ∼3^8^).

We next tested the reactivity profiles of the three designs and R4 relative to the substrates discussed above and hydrogen peroxide, again noting dramatic differences (Figure 3 and Table S4). 8H and R4 are the most efficient enzymes in terms of their turnover numbers (*k*_cat_), and the R4 affinity to some substrates is the greatest, as reflected in low *K*_M_. Substrate affinities, however, exhibit surprising diversity without establishing any of the enzymes as the best across the board. First, although 5H exhibits low *k*_cat_ values throughout, it exhibits more than an order of magnitude higher affinity for VA than the next best enzyme. Thus, despite the low catalytic turnover, its high VA affinity may make 5H well suited to lignin degradation in situations where VA concentrations are limiting. Second, 11H exhibits the highest affinity to H_2_O_2_ (by an order of magnitude relative to R4). Since H_2_O_2_ deactivates VPs and destabilizes proteins generally, a high affinity in this enzyme is beneficial in cases where hydrogen peroxide levels must be kept low to maintain activity in other enzymes. Third, 11H exhibits the highest affinity for manganese, by at least twofold (relative to R4). Last, the heme-dependent DMP affinity of 8H is at least 70-fold higher than that of the other VPs.

### Structural Basis for Functional Diversity in Designed VPs

**Figure 4:**
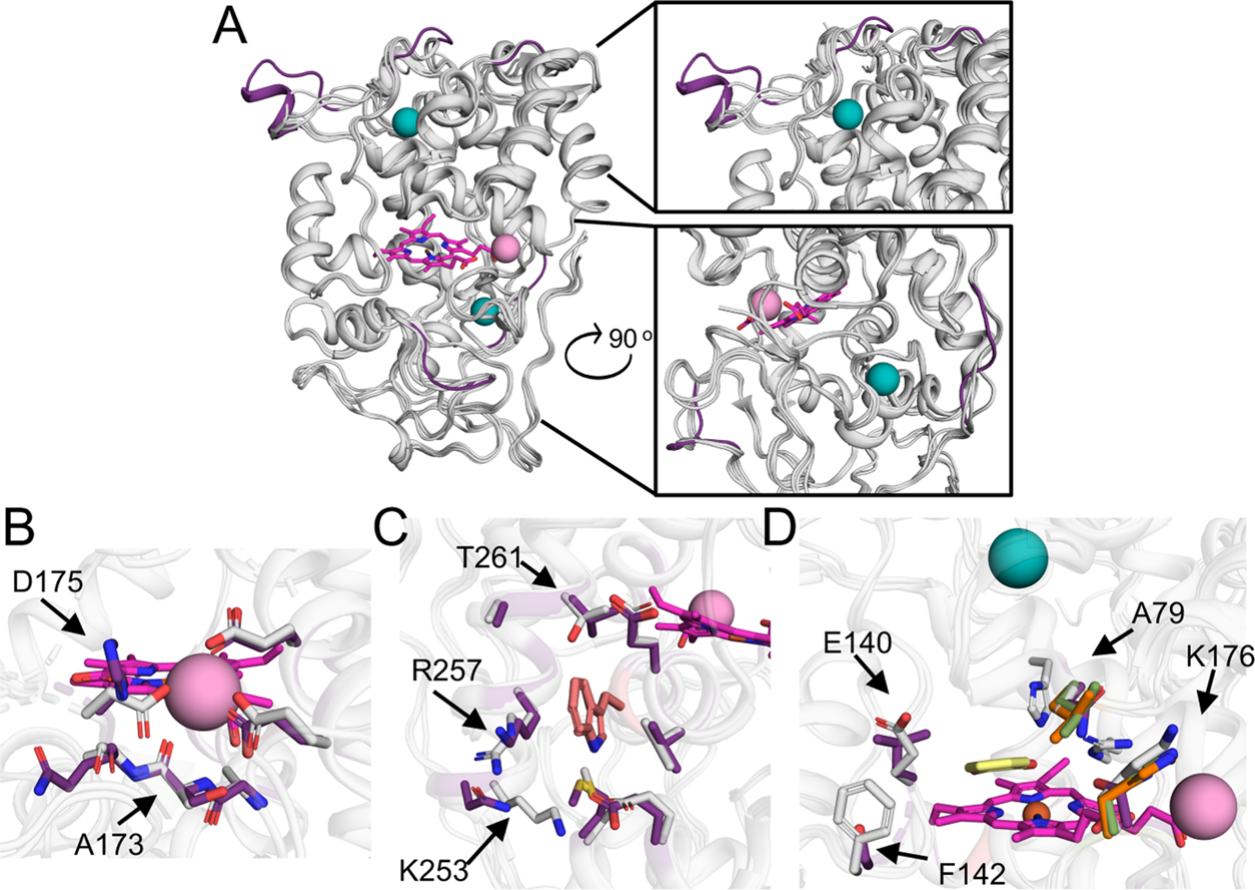
Structural basis for the different activity profiles in VPs. (A) AlphaFold2^23^ models of 5H, 8H, and 11H are superimposed onto the VPL crystallographic structure (PDB entry 3FJW). All VP backbones are presented in gray cartoons, VPL calcium and manganese ions are in teal and pink spheres, respectively, and the heme group in pink sticks in all panels. Backbone segments that are unique for 8H are colored in purple. (B) Manganese-oxidation site. VPL and 8H residues that chelate manganese and vicinal side chains are in gray and purple sticks, respectively. Significant variations are marked in arrows and their position identities and numbers are relative to the PDB entry 3FJW in all panels. (C) Reactive surface tryptophanyl site. Tryptophan is presented in salmon sticks. VPL and 8H residues in the tryptophan vicinity are presented in gray and purple sticks, respectively. (D) Access channel to the heme-oxidation site. Guaiacol (GUA), which is chemically similar to DMP, from a VPL crystallographic structure (PDB entry 4G05(32)) is in yellow sticks. VPL residues in the GUA vicinity are gray. Mutations relative to VPL are indicated in green, purple, and orange sticks for 5H, 8H, and 11H, respectively, and are marked by arrows.

8H was designed based on a VP from the Polyporales order, whereas the other designs, as well as R4, derive from the Agaricales order (and specifically, from genus *Pleurotus*). Thus, 8H diverges from the other VPs both in sequence (Tables S2 and S3) and in structure (Figure 4A). The most significant active-site differences between 8H and the other VPs are in a loop that chelates both heme and manganese (Figure 4B). Among the manganese-chelating residues, 8H exhibits an Asp → His mutation in position 175 (all position numbers relative to the PDB entry 3FJW), which was previously implicated in manganese oxidation.^27^ Additionally, mutation Ala173Ser near the manganese-binding site may also modify manganese-binding properties. These naturally occurring sequence changes likely explain the sharp decrease in the manganese-oxidation ability in 8H.

In addition to the manganese oxidation site, VPs oxidize substrates through a high-redox surface tryptophan site via a long-range electron-transfer mechanism, and a low-redox potential heme-dependent site. The four VPs we investigated exhibit large variations in kinetic constants in the high-redox surface site. Thus, despite the high accessibility of this site, the tryptophan environment’s chemical properties and molecular recognition play an important role in determining the reactivity at this site.^9^ 8H shows unique electrostatic and structural properties at this site as well. Whereas the other enzymes exhibit a conserved Lys and Arg that partially shield the tryptophan and a Thr residue on the opposite side, 8H presents an Asn, Lys, and Val, respectively (Figure 4C). Although 5H, 11H, and R4 share an identical sequence at the high-redox potential tryptophan active site, they exhibit significant changes in reactivity. These reactivity differences may stem from sequence changes along the electron-relay path that connects the tryptophan radical and the heme-dependent site in ways that remain to be clarified.

Finally, the models show that the heme pocket is conserved among all of the VPs. The only variations in its immediate surroundings are the manganese-bridging loop in 8H, a Ser → Ala substitution in position 168 in 5H, and hydrophobic-to-hydrophobic substitutions at positions 152, 234, and 262 in all VPs. Given these relatively minor sequence changes, we speculate that the ABTS and DMP activity differences stem from changes in substrate accessibility to the heme-binding pocket. Indeed, the heme access-channel loops exhibit dramatic variations, which influence the hydrophobicity of the pocket and its size. For example, 8H has a Leu instead of Glu in position 140, generating a hydrophobic access channel that may underlie the observed higher affinity for DMP; this mutation in 8H may also lead to a higher affinity for the product, explaining the low turnover number (Figure 4D). Previous mutation analyses demonstrated this position’s importance for substrate recognition and pH-dependent activity.^14,32^ We conclude that the significant diversity in the active sites and access channels among natural VPs underlies the large functional changes among the designs. These structure–activity relationships may inform future VP design efforts or help focus the search for natural VPs that exhibit unique functional features.

## Discussion

Lignocellulose is a heterogenous and stable material. To effectively decompose it, white-rot fungi deploy an arsenal of oxidoreductases that act synergistically, including through consortia comprising different fungal species.^8,10,36,37^ One of the major research goals in mobilizing lignocellulose for energy production is to deploy such an arsenal in a heterologous or cell-free setting.^38^ In the case of VPs, however, this goal is stymied by the lack of a common host for functional expression, thus demanding extensive enzyme engineering. The current enzyme-engineering approaches, however, are impossible to apply in cases in which the wildtype protein exhibits no functional expression in commonly used heterologous hosts as is the case for the VPs we targeted. Accordingly, VP engineering studies have focused on VPL and used directed evolution to isolate variants that exhibited higher expressibility, stability, and pH-dependent activity.^13−15^ We demonstrated that deep-learning *ab initio* structure prediction methods can be combined seamlessly with the PROSS protein-stability design method to deliver new VPs that add significant diversity to the arsenal of lignin oxidoreductases that have been characterized to date. Our structural analysis suggests that the observed functional diversity stems from the natural sequence diversity and that the design process serves only to expose and utilize this preexisting diversity. Notably, even the two closest paralogs (VP5 and VP11; 80% sequence identity) exhibit significant and potentially useful functional differences. Thus, the scope for exposing useful functional diversity through this modeling and design strategy is vast.

The designs exhibit several attractive features for use in a synergistic oxidoreductase cocktail. First, they are thermostable and relatively well expressed in *S. cerevisiae*. Second, they exhibit different pH tolerance and pH-dependent activity profiles that can be useful in different research or applied settings. Third, they show dramatic differences in substrate selectivity. Even with respect to a given substrate, the enzymes exhibit large differences in catalytic turnover and substrate affinity (*k*_cat_ and *K*_M_, respectively). By combining enzymes with high and low ligand affinity (and low and high turnover, respectively), one may generate a cocktail that can oxidize substrates quickly when substrate concentrations are high (through the high-turnover enzyme) and continue to oxidize substrates even when substrate concentrations are limiting (through the high-affinity enzyme), thus maximizing the cocktail’s effectiveness.^39,40^

Finally, we note the remarkably high accuracy of the new generation of deep-learning-based structure predictors. Prior to using the December 2020 version of trRosetta, we modeled the VPs using a previous version of trRosetta and using traditional homology-modeling software. The resulting structure models, however, demonstrated disordered regions, poor packing, abnormal geometries and did not exhibit features expected of a VP enzyme, such as the well-ordered heme-binding pocket (Figure S7). Therefore, we did not pursue them further. The most successful designs in our study comprised ∼40 mutations from the wildtype proteins, underscoring the high accuracy and reliability of the structure prediction and design methods we combined. Recently, even more reliable predictors have become available,^23,24^ though we note that in the specific case of VPs, they do not produce significantly improved models compared to the trRosetta models we used (Figure S7). Our work demonstrates that enzyme engineering can now be freed from the requirement of experimental structure determination through a streamlined pipeline that starts from *ab initio* modeling and continues to automated protein design. Accordingly, we have adapted the PROSS web server (https://PROSS.weizmann.ac.il/) to use AlphaFold2 models, automatically restricting the design to high-confidence regions in the models. Compared to previous VP engineering studies that required screening of thousands of variants,^13−15^ we screened fewer than 40 designs. Thus, model-based enzyme design may dramatically increase the scope of computational design methods and address the growing need in many fields to generate functional, stable, and efficient enzyme repertoires or pathways in one shot.^41^

## Supporting Information Available

A detailed description of the computational methods, materials, and experimental procedures; amino acid sequences of the wildtype and characterized designs; Tables S1–S4 include origins of the selected sequences, their designed mutational loads and their homology, and report the full kinetic data; and Figures S1–S7 show the structural model accuracy, PROSS mutations, and full activity and stability profiles of the most successful designs (PDF)
