# Supplementary material for: Stable and Functionally Diverse Versatile Peroxidases Designed Directly from Sequences
Source: J Am Chem Soc. 2022 Feb 18;144(8):3564–71. doi: 10.1021/jacs.1c12433 (PMC8895400; doi:10.1021/jacs.1c12433)
Supplement: Supplementary file 1 — ja1c12433_si_001.pdf [file ja1c12433_si_001.pdf]

Supporting information for:

Stable and functionally diverse versatile peroxidases designed directly from sequence

Shiran Barber-Zucker[1], Vladimir Mindel[1], Eva Garcia-Ruiz[2], Jonathan J. Weinstein[1], Miguel Alcalde[2] and Sarel J. Fleishman[1]*

[1] Department of Biomolecular Sciences, Weizmann Institute of Science, Rehovot 7600001, Israel

[2] Department of Biocatalysis, Institute of Catalysis, CSIC, Cantoblanco, Madrid 28094, Spain

*sarel@weizmann.ac.il

Table of Contents

Computational methods 2

Materials and experimental procedures 3

Amino acid sequences of characterized VP designs 8

Table S1. Selected VP origins, protein lengths and number of mutations in each design 9

Table S2. Selected VPs homology 10

Table S3. Most-active VP designs sequence homology (in percentage) 10

Table S4. Kinetic parameters with various substrates 11

Figure S1. Accuracy of trRosetta models 12

Figure S2. Sequence alignment of 5H, 8H and 11H to their wildtype progenitor 13

Figure S3. Examples of PROSS mutations in VP designs 14

Figure S4. Thermal stabilities of selected VP designs 14

Figure S5. pH stabilities of selected VP designs 15

Figure S6. pH activity profiles of selected VP designs with various substrates 15

Figure S7. 2020's evolution of deep-learning based *ab initio* structure prediction methods 16

References 17

Computational Methods

VP sequence collection. VP sequences were extracted from three databases: MycoCosm (fungal genome database[1]), RedoxiBase (oxidoreductases database[2]), and fPoxDB (fungal peroxidases database[3]). Signal peptide sequences were identified using SignalP-5.0[4] and were removed from the relevant sequences. A multiple sequence alignment was generated using MUSCLE[5] based on all unique sequences, and a phylogenetic tree was inferred using the Maximum Likelihood method and JTT matrix-based model through MEGA[6,7]. For each clade, a consensus sequence was generated using EMBOSS Cons[8], and the VP sequence with the highest similarity to the consensus was chosen for further analysis.

trRosetta structure modeling. The structures of all the selected sequences were calculated using the trRosetta structure prediction algorithm (December 2020 version[9,10]) through the Robetta server (https://robetta.bakerlab.org/). The models were visually inspected, and ones exhibiting poor parameters were eliminated; for instance, models that did not adopt the expected VP fold or in which the catalytic site diverged from expectation. Finally, twelve diverse sequences (eleven sequences from the analysis above and VPL), with 51-81% identity between each sequence pair, were selected for further modeling and design (Table S1 & S2).

PROSS stability design calculations. For each sequence, the best-calculated model was subjected to PROSS design[11,12]. VPL was also designed by PROSS starting from its crystal structure (PDB entry: 3FJW). Since trRosetta does not predict ligands or ions, structural information from the VPL structure was used to determine the putative binding sites of the heme, manganese, and two structural calcium ions, as well as of the active tryptophan, and to limit mutations in these sites (design in all residues within 4 Å of any of the ligands atoms was disallowed). Structural inspection of the models was used to disallow design in positions that showed structural inconsistency in the best five models calculated by trRosetta, and analysis of the designs was used to exclude mutations in positions that are in contact with such positions. Automated filtering of such positions based on AlphaFold2 models is now enabled on the PROSS web server (https://PROSS.weizamann.ac.il). Further, any mutations that might change native N-glycosylation patterns were dismissed from the designs.

AlphaFold2 modeling. The sequences of 5H, 8H and 11H were structurally modeled by the AlphaFold2 algorithm[13], using the AlphaFold Colab notebook:
https://colab.research.google.com/github/deepmind/alphafold/blob/main/notebooks/AlphaFold.ipynb

Materials and Experimental Procedures

Reagents. The protease-deficient *S. cerevisiae* strain BJ5465 (α ura3-52 trp1 leu2Δ1 his3Δ200 pep4::HIS3 prb1Δ1.6R can1 GAL) was obtained from LGCPromochem (Barcelona, Spain). The uracil-independent and ampicillin resistance shuttle vector pJRoC30 was obtained from the California Institute of Technology (Caltech, Pasadena, CA). The α-factor prepro-leader sequence and all the VP genes sequences were ordered from Twist Biosciences (San Francisco, CA). The BamHI and XhoI restriction enzymes were ordered from New England Biolabs (NEB, Rehovot, Israel). ABTS, VA, RB5, and the *S. cerevisiae* transformation kit, were purchased from Sigma–Aldrich (Rehovot, Israel). DMP was purchased from Acros Organic (Geel, Belgium) and hemoglobin from bovine erythrocytes from EMD Millipore Corp (Billerica, MA, USA).

Cloning of VP genes. Cloning of all VP genes was performed by using the *S. cerevisiae* homologous recombination machinery[14]. pJRoC30-AAO (aryl-alcohol oxidase) expression shuttle vector previously constructed in the Alcalde lab[15] was digested with BamHI and XhoI restriction enzymes to remove the signal peptide and the AAO gene constructed within it. The α-factor prepro-leader DNA sequence, that was used in a previous directed evolution campaign of VPL (including additional restriction site in its 3' that encodes for Glu-Phe dipeptide in the N-terminal of the mature proteins)[16], was ordered as a gene fragment with 40 bp overlap to the linearized plasmid, and the VP genes were ordered each with 40 bp overlap to the signal peptide sequence, and to the linearized plasmid. The design of the 40 bp overlapping regions between the three fragments (plasmid, signal peptide, VP genes) allowed the recombination machinery of the protease-deficient *S. cerevisiae* strain BJ5465 to drive the fusion of the three DNA elements after transformation, and to form the pJRoC30-SignalPeptide-VPgene expression shuttle vector. pJRoC30-VPL-WT, -R4, and -2-1B were constructed previously in the Alcalde lab[16]. All *S. cerevisiae*-transformed cells were plated in synthetic complete (SC) drop-out plates, and in each plate, selected colonies were picked and sequenced to verify the correct assembly and gene sequence.

Culture media. Minimal medium is composed of 6.7 g/L yeast nitrogen base, 1.92 g/L amino acids supplements (yeast synthetic drop-out medium supplements without uracil), 2% raffinose and 25 mg/L chloramphenicol. VPs expression medium is composed of YP x1.11 medium (22.2 g/L bacto peptone and 11.1 g/L yeast extract), 67 mM KH_2PO_4 buffer at pH 6.0, 25 g/L ethanol, 22.2 g/L D-galactose, 500 mg/L bovine hemoglobin, 1 mM $CaCl_2$ and 25 mg/L chloramphenicol. SC drop-out plates are composed of 6.7 g/L yeast nitrogen base, 1.92 g/L amino acids supplements (yeast synthetic drop-out medium supplements without uracil), 2% glucose, 20 g/L Bacto agar and 25 mg/L chloramphenicol.

Screening for active variants. A colony from each *S. cerevisiae* clone containing the parental or mutant VP gene was picked from an SC drop-out plate, inoculated in 2 mL minimal medium in a 14 mL culture tube, and incubated for 48 hours at 30 °C and 225 rpm. An aliquot of cells was removed and used to inoculate 2 mL of minimal medium in a new 14 mL culture tube to an OD_{600nm} of 0.25-0.30, under the same

conditions. The cells completed two growth phases (8-10 hours, reaching OD_{600nm} ~ 1), then the expression medium (2.7 mL) was inoculated with 0.3 mL of the pre-culture in a new 14 mL culture tube (OD_{600nm} ~ 0.1). Cells were incubated for further ~38-40 hours at 30 °C and 225 rpm and then centrifuged at 4000 g for 20 min at 4°C. The supernatant was removed into new tubes for further analysis. The expression protocol ran in triplicate, with an empty vector (containing only the signal peptide sequence) and the VPL-R4 and -2-1B variants as negative and positive controls, respectively. An ABTS-based colorimetric assay was conducted to assess the variants' activity: 20 μL of supernatant were transferred into activity 96 plates (Greiner Bio-One GmbH, Kremsmünster, Austria), and then, 180 μL of the reaction mixture were added to each row in the plate, and absorption at 418 nm was recorded immediately in a kinetic mode in a plate-reader at 25 °C (Citation5 or Synergy HTX plate readers, Bio-Tek, Bad Friedrichshall, Germany). The reaction mixture contained 100 mM citrate–phosphate buffer (pH 4.0), 2 mM ABTS and 0.1 mM H_2O_2. The activities were recorded in triplicate.

Small scale production of active variants. A colony from each *S. cerevisiae* clone containing the parental or mutant VP gene was picked from an SC drop-out plate, inoculated in 2 mL minimal medium in a 14 mL culture tube, and incubated for 48 hours at 30 °C and 225 rpm. An aliquot of cells was removed and used to inoculate 2.7 mL of minimal medium in a new 14 mL culture tube to an OD_{600nm} of 0.25-0.30, under the same conditions. The cells completed two growth phases (8-10 hours, reaching OD_{600nm} ~ 1), then the expression medium (9 mL) was inoculated with 1 mL of the pre-culture in a 50 mL Falcon tube (OD_{600nm} ~ 0.1). Cells were incubated for further ~60 hours at 30 °C and 225 rpm and then centrifuged at 4000 g for 20 min at 4°C. The supernatant was removed into new tubes for further characterization of VPs in supernatant.

Thermostability assay (T_{50}). Aliquots of 30 μL of selected variants' supernatant at appropriate dilutions (with 20 mM piperazine pH=5.5, buffer A, to achieve linear response in kinetic mode measurements in activity reads) were used for each incubation temperature. The samples were incubated for 10 or 15 minutes in a thermocycler pre-heated to a specific temperature (every 5 °C in a gradient scale ranging from 25 to 80 °C) and then removed and chilled on ice for 10 min. Thereafter, samples were removed from ice and incubated for at least 5 min at room temperature. Activity at each temperature was measured using the ABTS-based colorimetric assay described above and was normalized to the activity at 25 °C for residual activity calculations. All incubations and activity assays were conducted in triplicate. T_{50} values were calculated by sigmoidal fit to the T_{50} data of 5H, 8H, 11H and R4 (15 minutes incubation).

Kinetic thermostability ($t_{1/2}$). Aliquots of 30 μL of selected variants' supernatant at appropriate dilutions (with buffer A, to achieve linear response in kinetic mode measurements in activity reads) were used for each incubation time point. The samples were incubated in a thermocycler (S1000™ thermocycler, Bio-Rad, Rishon LeZion, Israel) pre-heated to 60 °C or 65 °C, and removed at different times (after 0, 2, 5, 7, 10, 15, 20, 30, 45, 60, 90 and 120 min), chilled out on ice for 10 min and further incubated at room temperature at least for 10 min. Activity at each time point was measured using the ABTS-based

colorimetric assay described above and was normalized to the activity at time 0 for residual activity calculations. All incubations and activity assays were conducted in triplicate.

pH stability. Supernatants of the selected variants were diluted to reach a final concentration of 100 mM citrate-phosphate-borate buffer at pH ranging from 2-9. Aliquots of 20 µL were removed at different times (time 0, 4, 25, 50, 75 and 165 hours) and measured in the regular ABTS-based colorimetric assay described above, but here in the presence of 180 µL of the following reaction mixture: 111.11 mM citrate-phosphate-borate buffer (pH 4.0), 2.22 mM ABTS and 0.111 mM H_2O_2. For pH 2, an additional experiment was conducted, under the same procedure but with aliquots being removed at different time points (time 0, 10, 20, 30, 45, 60, 75, 90, 120, and 150 min). For the assay in pH range 2-9, activities were normalized to the activity at time 0 in pH=3, for residual activity calculations. All incubations and activity assays were conducted in triplicate.

VPs production and purification. A colony from *S. cerevisiae* clone containing the VP gene (5H, 8H, 11H, VPL_R4, VPL_2-1B) was picked from an SC drop-out plate, inoculated in 25 mL minimal medium in a 250 flask, and incubated for 48 hours at 30 °C and 225 rpm. An aliquot of cells was removed and used to inoculate 100 mL of minimal medium in a 1 L flask to an OD_{600nm} of 0.25-0.30, under the same conditions. The cells completed two growth phases (8-10 hours, reaching OD_{600nm} ~ 1-1.5), then the expression medium (450 mL) was inoculated with 50 mL of the pre-culture in a 2 L flask (OD_{600nm} ~ 0.1). Cells were incubated for a further ~60 hours at 30 °C and 225 rpm. Thereafter, cells were centrifuged at 6000 g for 15 min at 4°C, and the supernatant was collected and filtered with a 0.2 µm filter bottle.

Filtrates were subjected to fractional precipitation with ammonium sulfate in two steps: a first cut of 50%, followed by centrifugation and elimination of the precipitates, and a second cut of 70%. Buffer A was used to dissolve the pellet of the second cut, and the dissolved protein solution was shaken overnight at 4 ^{o}C for maximal recovery. The dissolved fraction was then centrifuged, filtrated, concentrated, and subjected to overnight dialysis against buffer A. Filtered fractions of the VP proteins after dialysis were uploaded into a HiTrap^{TM} Q HP Column (GE Healthcare Bio-Sciences AB, Uppsala, Sweden) pre-equilibrated with buffer A, through ÄKTA pure protein purification system (GE Healthcare Bio-Sciences AB). Proteins were eluted in a two-step linear gradient from 0 to 1 M NaCl, at a flow rate of 1 mL/min: the first phase of 0-25 % over 15 column volumes (75 min) and second phase of 25-100 % over 2 column volumes (10 min). The fractions of the peak with the highest VP activity (and absorption at 407 nm) were pooled, concentrated, and dialyzed against 20 mM piperazine buffer pH=5.5 and 150 mM NaCl (buffer B). Protein fractions were then uploaded onto a Superdex 75 Increase 10/300 GL (GE Healthcare Bio-Sciences AB) through the ÄKTA pure system pre-equilibrated with buffer B. The fractions of the peak with the highest VP activity (and absorption at 407 nm) were pooled and dialyzed against buffer A. Pure protein samples were stored at 4 °C. Protein concentration was determined using the BCA assay with bovine serum albumin as a standard. The obtained Reinheitszahl values (Rz: $Abs_{407\ nm}/Abs_{280\ nm}$), which indicate for the purity of peroxidases, were 1 for 8H (due to high tryptophan content and therefore extinction coefficient at 280 nm) and above 2 for 5H, 11H, R4 and 2-1B.

Hydrogen peroxide stability. Purified 5H, 8H, 11H, R4 and 2-1B at 250 nM concentration (diluted with buffer A, to achieve linear response in kinetic mode measurements in activity reads) were incubated for 50 minutes at room temperature with 750 µM H_2O_2 (1:3,000 molar ratio). An aliquot of 20 µL was removed at times 0, 3, 7, 12, 18, 28, 38 and 48 minutes. Activity was immediately measured using the ABTS-based colorimetric assay described above and was normalized to the activity at time zero for residual activity calculations. All incubations and activity assays were conducted in triplicate.

pH activity profiles. For purified 5H, 8H, 11H, and R4, 20 µL protein samples (diluted in buffer A) were transferred into activity 96 plates (in the case of VA and $MnSO_4$, UV-Star plates; Greiner Bio-One GmbH, Kremsmünster, Austria) and then, 180 µL of the reaction mixture were added to each row in the plate, and absorption at the appropriate wavelength (substrate-dependent) was recorded immediately in a kinetic mode in a plate-reader at 25 °C. The reaction mixtures contained a specific substrate in 100 mM citrate–phosphate-borate buffer (pH 2, 3, 3.5, 4, 5, 6, 7, 8) and 0.1 mM H_2O_2. The activities were recorded in triplicate. The following substrate concentrations and absorption wavelengths were used: ABTS: 2 mM, 418 nm; DMP: 5 mM, 469 nm; RB5: 0.05 mM, 598 nm; VA: 30 mM, 310 nm. For manganese, the reaction mixtures contained 60 mM $MnSO_4$ in 100 mM sodium tartrate buffer (pH 3, 3.5, 4, 4.5, 5) and 0.1 mM H_2O_2, and the Mn^{3+}-tartrate complex absorption was read at 238 nm. For each protein and substrate, the activities were normalized to the activity at optimal pH for residual activity calculations. Each activity assay was conducted in triplicate.

Kinetic parameters. Steady-state kinetics were determined for 5H, 8H, 11H and R4, by measuring the activity (initial rates) in increasing concentrations of the substrate. The K_M and k_{cat} values for RB5, VA and Mn^{2+} were calculated by fitting the results to the Michaelis-Menten model ($V_0 = k_{cat}[E][S] / (K_M + [S])$). For ABTS and DMP, using semi-logarithmic [S]-axis scale, double hyperbolic curves could be obtained, implying two oxidation sites (of high and low efficiency, in the µm and mm ranges). Each of the curve regions were fitted separately to the Michaelis-Menten model, which enabled the calculation of the two sets of kinetic constants. 20 µL purified protein samples (diluted in buffer A to appropriate concentration, [E]<<[S]) were transferred into activity 96 plates (in the case of VA and $MnSO_4$, UV-Star plates) and then, 180 µL of the reaction mixture were added to each row in the plate, and absorption at the appropriate wavelength (substrate-dependent) was recorded immediately in a kinetic mode in a plate-reader at 25 °C. The reaction mixtures contained substrates at varying concentrations, in 100 mM citrate–phosphate-borate buffer at optimum pH (for manganese, sodium tartrate buffer was used) and optimum H_2O_2 concentration (0.4 mM for 5H, 0.2 mM for 8H, 0.1 mM for 11H and 1 mM for R4; approximately double of the K_M values were used to gain high activity with minimal inhibition effect). H_2O_2 kinetics was measured using 2 mM (5H, 11H and R4) or 3 mM (8H) ABTS in 100 mM citrate–phosphate-borate buffer at optimum pH for ABTS activity. The following molar extinction coefficients were used to calculate the substrate/product concentration: ABTS, $\varepsilon_{418\ nm} = 36{,}000\ M^{-1}\ cm^{-1}$; DMP, $\varepsilon_{469\ nm} = 27{,}500\ M^{-1}\ cm^{-1}$; RB5, $\varepsilon_{598\ nm} = 30{,}000\ M^{-1}\ cm^{-1}$; VA, $\varepsilon_{310\ nm} = 9300\ M^{-1}\ cm^{-1}$; Mn^{3+}-tartrate, $\varepsilon_{238\ nm} = 6500\ M^{-1}\ cm^{-1}$. All activities were recorded in triplicate and the average velocity was used for the kinetic constants calculations.

Expression level calculations. Enzyme concentrations, [E], were extracted from the Michaelis-Menten equation ($V_0 = k_{cat}[S][E] / (K_M + [S])$), where V_0 are the initial rates observed during the screening experiments (means of biological triplicates) and [S] is the ABTS concentration at this experiment. The kinetic constants correspond to the calculated K_M and k_{cat} for ABTS as reported in Table S4 (of the low-efficiency site that dominates the reaction at the used ABTS concentration). The activity assay was performed at pH=4.0, therefore the initial activities were normalized to the activity at optimal pH (using the data from pH-dependent activity assay; Figure S5).

Amino acid sequences of characterized VP designs:

5WT:
VSLPQKRATCAGGQVTANAACCVLFPLMEDLQKNLFDDGACGEDAHEALRLTFHDAIGFSPSRGVMGGADGSVITFS
DTEVNFPANLGIDEIVEAEKPFLARHNISAGDLVHFAGTLAVTNCPGAPRIPFFLGRPPAKAASPIGLVPEPFDTIT
DILARMDDAGFVSVEVVWLLSAHSVAAADHVDETIPGTPFDSTPNLFDSQIFIETQLRGISFPGTGGNHGEVQSPLK
GEMRLQSDHLFARDDRTSCEWQSMTNDQQKIQDRFSDTLFKMSMLGQNQDAMIDCSDVIPVPAALVTKPHLPAGKSK
TDVEQACATGAFPALGADPGPVTSVPRVPPA

5H:
VSLPQKRATCSGGQTTSNEACCVLFDLMEDLQKNLFDGGQCGEQAHEALRLTFHDAIGFSPSRGVMGGADGSVITFS
DIETNFPANLGIDDIVEAEKSFLQRHNISAGDLVHFAATLAVTNCPGAPRIPFFLGRPPATAPSPPGLVPEPFDSVT
DILARMADAGFSPVEVVWLLSAHSVAAADHVDPTIPGTPFDSTPNLFDSQFFIETQLRGTTFPGTGGNPGEVKSPLP
GEMRLQSDHLFARDPRTACEWQSMVNDQQKIQDRFRDTLFKMSMLGQNQDDMIDCSDVIPVPPPLTTKPHLPAGKSK
TDVEQACATAPFPTLPADPGPPTSVPPVPPA

8WT:
AVPRMGKRATCSNGKTVNNDACCVWFDVLDDIQENLFHGGQCGEDAHEALRLTFHDAIGFSPALTAAGQFGGGGADG
SIIAHSDVELTYPANNGVDEIVEASRPIAIKHNVSFGDFIQFAGAVGTANCNGGPQLSFFAGRSNDSQPAPPNLVPL
PSDSADSILSRFSDAGFDAVEVVWLLVSHTVGSQHTVDPSIPGAPFDSTPSDFDAQFFVETMLNGTLVPGNGLQDGE
VNSPYPGEFRLQSDFALSRDSRTACEWQKMIADRANMLAKFEGVMLKMSLLGFDQSALTDCSDVIPTATGTVQDPFL
PAGLTVDDLQPACSSSAFPTVSTVAGAATSIPAVPMDS

8H:
AVPPSGKRATCSNGKTVNNDACCVWFDVLDDIQTNLFHGGQCGEDAHEALRLTFHDAIAFSPALWAQGQFGGGGADG
SIIAHSDIELTYPANNGIDEIVEASRHIAQKHNVSFGDFIQFAGAVGVANCNGGPQLPFFAGRPNPSQPAPPNLVPL
PSDSADQILARFADAGFSAVEVVWLLVSHTVGSQHTVDPSIPGAPFDSTPSDFDAQFFVETMLNGTLVPGNGLQQGE
VNSPYPGEFRLQSDFLLARDPRTACEWQKMIADQDNMQSKFAAVMLKMSLLGFDQSSLIDCSDVIPTPPGTVQDPFL
PAGLTVDDLQPACSDSPFPTVPTVPGPATSIPPVPMDS

11WT:
VTLPQKRATCAGGQVTANAACCVLFPILEDLQQNLFDGGECGEEVHESLRLTFHDAIGFSPTKGGGGADGSVLTFSD
PEVNFPANLGIDEIVEAQKPFLARHNISAGDLVQFAGALGVSNCPGAPRIPFFLGRPPAKAASPIGLVPEPFDTVTD
ILDRMGDAGFAAVEVVWLLSSHTIAAADHVDESIPGTPFDSTPSIFDSQFFIETQLRGTSFPGSGGNHGEVESPLAG
EIRLQSDHLLARDSRTSCEWQSMVDNMPKIQNRFAATMLKMSLLGQNQADLIDCSDVIPTPPALVGKAHLPAGKVQS
DVEQACATTPFPAIAADPGPVTAVPPVPPS

11H:
VTLPQKRATCSGGQTTSNAACCVLFDLRDDLQKNLFDGGQCGEEVHESLRLTFHDAIGFSPTKGGGGADGSVLIFSD
TELNFPANLGIDEIVEAQKPFLQRHNISAGDLVQFAGALGVSNCPGAPRIPFFLGRPPATAPSPDGLVPEPFDSVDD
ILARMADAGFSPVEVVWLLSSHTIAAADHVDPTIPGTPFDSTPSIFDSQFFIETQLRGTLFPGTGGNPGEVESPLPG
EIRLQSDHLLARDPRTACEWQSMVDNMPKIQNRFAATMLKMSLLGQNVRDLIDCSDVIPTPPPLVGTAHLPAGKTQS
DVEQACATTPFPTIPADPGPVTSVPPVPPS

Supplementary Tables

Table S1. Selected VPs origins, protein lengths and number of mutations in each design

Name	Species	Protein length	# mut. H	# mut. M	# mut. L
VP8	*Ganoderma* sp. 10597_SS1	346	38	25	12
VP2	*Dichomitus squalens*	353	29	18	11
VP7	*Trametes versicolor*	338	49	22	13
VP4	*Lentinus tigrinus* ALCF2SS1-6s	345	40	21	8
VP3	*Gelatoporia subvermispora B*	338	38	21	15
VP10	*Pleurotus* sp. Florida	344	33	22	11
VPL	*Pleurotus eryngii* (VPL2)	331	39	23	16
VP5	*Pleurotus ostreatus*	339	43	27	18
VP11	*Pleurotus ostreatus*	338	38	23	13
VP9	*Ganoderma* sp. 10597 SS1	335	30	19	11
VP1	*Bjerkandera adusta*	340	27	14	7
VP6	*Physisporinus* sp. PF18	337	25	15	8

mut refers to the number of mutations in each designed variant: H – high mutational load, M – medium mutational load, L – low mutational load.

Table S2. Selected VP sequences homology (in percentage)

Name	VP8	VP2	VP7	VP4	VP3	VP10	VPL	VP5	VP11	VP9	VP1	VP6
VP8	100	77	76	75	52	57	57	52	55	53	57	55
VP2		100	80	81	51	58	58	53	58	52	57	55
VP7			100	81	53	60	60	55	60	54	60	57
VP4				100	83	59	59	54	58	55	59	56
VP3					100	64	66	63	66	66	69	69
VP10						100	73	66	76	68	68	72
VPL							100	69	78	68	70	71
VP5								100	80	61	61	64
VP11									100	66	68	72
VP9										100	71	74
VP1											100	76
VP6												100

Table S3. Most-active VP designs sequences homology (in percentage)

Name	VPL	5H	8H	11H
VPL	100	74	66	81
5H		100	60	83
8H			100	64
11H				100

Table S4. Kinetic parameters with various substrates

Substrate	Kinetic constants	5H	8H	11H	R4
ABTS (high efficiency)	K_M (µM)<br>k_{cat} (sec^{-1})<br>k_{cat} / K_M (sec^{-1} mM^{-1})	1.9 ± 0.4<br>1.5 ± 0.1<br>763 ± 151	5.0 ± 0.8<br>7.8 ± 0.3<br>1550 ± 260	6.3 ± 0.7<br>2.0 ± 0.1<br>314 ± 37	0.39 ± 0.08<br>1.3 ± 0.1<br>3,300 ± 700
ABTS (low efficiency)	K_M (µM)<br>k_{cat} (sec^{-1})<br>k_{cat} / K_M (sec^{-1} mM^{-1})	765 ± 96<br>33.2 ± 1.8<br>43.4 ± 5.9	795 ± 96<br>28.2 ± 0.9<br>35.4 ± 4.4	1,100 ± 90<br>11.2 ± 0.3<br>10.3 ± 0.9	987 ± 107<br>400 ± 21<br>405 ± 49
DMP (high efficiency)	K_M (µM)<br>k_{cat} (sec^{-1})<br>k_{cat} / K_M (sec^{-1} mM^{-1})	85.7 ± 16.9<br>1.2 ± 0.1<br>14.4 ± 3.0	22.9 ± 1.8<br>2.2 ± 0.1<br>94.6 ± 8.2	168 ± 10<br>1.91 ± 0.04<br>11.4 ± 0.7	1.4 ± 0.3<br>0.74 ± 0.05<br>542 ± 113
DMP (low efficiency)	K_M (µM)<br>k_{cat} (sec^{-1})<br>k_{cat} / K_M (sec^{-1} mM^{-1})	27,700 ± 7,100<br>5.4 ± 0.6<br>0.19 ± 0.05	164 ± 12<br>3.6 ± 0.1<br>22.1 ± 1.6	11,400 ± 2,000<br>6.3 ± 0.4<br>0.55 ± 0.10	16,100 ± 1,700<br>132 ± 7<br>8.2 ± 1.0
Mn^{2+}	K_M (µM)<br>k_{cat} (sec^{-1})<br>k_{cat} / K_M (sec^{-1} mM^{-1})	3,500 ± 200<br>9.1 ± 0.1<br>2.6 ± 0.1	11,300 ± 1,400<br>0.64 ± 0.03<br>0.06 ± 0.01	289 ± 34<br>19.7 ± 0.5<br>68.1 ± 8.2	523 ± 28<br>78.3 ± 1.1<br>150 ± 8
VA	K_M (µM)<br>k_{cat} (sec^{-1})<br>k_{cat} / K_M (sec^{-1} mM^{-1})	158 ± 27<br>0.52 ± 0.02<br>3.3 ± 0.6	10,100 ± 400<br>5.5 ± 0.1<br>0.55 ± 0.02	2,000 ± 100<br>1.38 ± 0.03<br>0.68 ± 0.05	7,400 ± 1,000<br>5.4 ± 0.4<br>0.7 ± 0.1
RB5	K_M (µM)<br>k_{cat} (sec^{-1})<br>k_{cat} / K_M (sec^{-1} mM^{-1})	2.9 ± 1.0<br>0.18 ± 0.04<br>62.3 ± 25.2	17.3 ± 1.7<br>1.1 ± 0.1<br>65.0 ± 7.2	2.0 ± 0.2<br>0.30 ± 0.01<br>152 ± 20	1.3 ± 0.1<br>0.63 ± 0.02<br>487 ± 51
H_2O_2	K_M (µM)<br>k_{cat} (sec^{-1})<br>k_{cat} / K_M (sec^{-1} mM^{-1})	197 ± 20<br>20.3 ± 0.8<br>103 ± 11	89.7 ± 12.9<br>12.1 ± 0.6<br>135 ± 21	38.9 ± 6.9<br>7.0 ± 0.5<br>180 ± 35	457 ± 53<br>230 ± 9<br>503 ± 61

Supplementary Figures

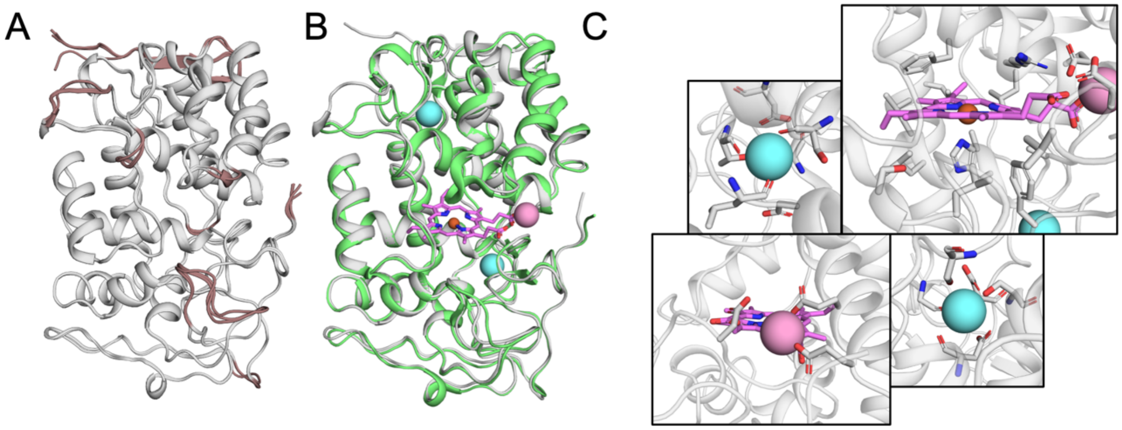

Figure S1. Accuracy of trRosetta models. (A) Four most reliable (top-ranked) models of a representative VP (VP5, gray), superimposed onto one another, demonstrate that most of the protein structural elements converge and small discrepancies occur only in peripheral loops (brown). (B) Best model of VP5 (gray) overlapped onto wild type VPL crystal structure (PDB entry 3FJW; light green). VPL calcium and manganese ions are presented in blue and pink spheres, respectively, and the heme group in pink sticks. (C) Close-up look onto VP5 residues that face all ligands and ions.

```
5WT   VSLPQKRATCAGGQVTANAACCVLFPLMEDLQKNLFDDGACGEDAHEALRLTFHDAIGFS   60
5H    VSLPQKRATCSGGQTTSNEACCVLFDLMEDLQKNLFDGGQCGEQAHEALRLTFHDAIGFS   60

5WT   PSRGVMGGADGSVITFSDTEVNFPANLGIDEIVEAEKPFLARHNISAGDLVHFAGTLAVT  120
5H    PSRGVMGGADGSVITFSDIETNFPANLGIDDIVEAEKSFLQRHNISAGDLVHFAATLAVT  120

5WT   NCPGAPRIPFFLGRPPAKAASPIGLVPEPFDTITDILARMDDAGFVSVEVVWLLSAHSVA  180
5H    NCPGAPRIPFFLGRPPATAPSPPGLVPEPFDSVTDILARMADAGFSPVEVVWLLSAHSVA  180

5WT   AADHVDETIPGTPFDSTPNLFDSQIFIETQLRGISFPGTGGNHGEVQSPLKGEMRLQSDH  240
5H    AADHVDPTIPGTPFDSTPNLFDSQFFIETQLRGTTFPGTGGNPGEVKSPLPGEMRLQSDH  240

5WT   LFARDDRTSCEWQSMTNDQQKIQDRFSDTLFKMSMLGQNQDAMIDCSDVIPVPAALVTKP  300
5H    LFARDPRTACEWQSMVNDQQKIQDRFRDTLFKMSMLGQNQDDMIDCSDVIPVPPPLTTKP  300

5WT   HLPAGKSKTDVEQACATGAFPALGADPGPVTSVPRVPPA   339
5H    HLPAGKSKTDVEQACATAPFPTLPADPGPPTSVPPVPPA   339
```

```
8WT   AVPRMGKRATCSNGKTVNNDACCVWFDVLDDIQENLFHGGQCGEDAHEALRLTFHDAIGF   60
8H    AVPPSGKRATCSNGKTVNNDACCVWFDVLDDIQTNLFHGGQCGEDAHEALRLTFHDAIAF   60

8WT   SPALTAAGQFGGGGADGSIIAHSDVELTYPANNGVDEIVEASRPIAIKHNVSFGDFIQFA  120
8H    SPALWAQGQFGGGGADGSIIAHSDIELTYPANNGIDEIVEASRHIAQKHNVSFGDFIQFA  120

8WT   GAVGTANCNGGPQLSFFAGRSNDSQPAPPNLVPLPSDSADSILSRFSDAGFDAVEVVWLL  180
8H    GAVGVANCNGGPQLPFFAGRPNPSQPAPPNLVPLPSDSADQILARFADAGFSAVEVVWLL  180

8WT   VSHTVGSQHTVDPSIPGAPFDSTPSDFDAQFFVETMLNGTLVPGNGLQDGEVNSPYPGEF  240
8H    VSHTVGSQHTVDPSIPGAPFDSTPSDFDAQFFVETMLNGTLVPGNGLQQGEVNSPYPGEF  240

8WT   RLQSDFALSRDSRTACEWQKMIADRANMLAKFEGVMLKMSLLGFDQSALTDCSDVIPTAT  300
8H    RLQSDFLLARDPRTACEWQKMIADQDNMQSKFAAVMLKMSLLGFDQSSLIDCSDVIPTPP  300

8WT   GTVQDPFLPAGLTVDDLQPACSSSAFPTVSTVAGAATSIPAVPMDS   346
8H    GTVQDPFLPAGLTVDDLQPACSDSPFPTVPTVPGPATSIPPVPMDS   346
```

```
11WT  VTLPQKRATCAGGQVTANAACCVLFPILEDLQQNLFDGGECGEEVHESLRLTFHDAIGFS   60
11H   VTLPQKRATCSGGQTTSNAACCVLFDLRDDLQKNLFDGGQCGEEVHESLRLTFHDAIGFS   60

11WT  PTKGGGGADGSVLTFSDPEVNFPANLGIDEIVEAQKPFLARHNISAGDLVQFAGALGVSN  120
11H   PTKGGGGADGSVLIFSDTELNFPANLGIDEIVEAQKPFLQRHNISAGDLVQFAGALGVSN  120

11WT  CPGAPRIPFFLGRPPAKAASPIGLVPEPFDTVTDILDRMGDAGFAAVEVVWLLSSHTIAA  180
11H   CPGAPRIPFFLGRPPATAPSPDGLVPEPFDSVDDILARMADAGFSPVEVVWLLSSHTIAA  180

11WT  ADHVDESIPGTPFDSTPSIFDSQFFIETQLRGTSFPGSGGNHGEVESPLAGEIRLQSDHL  240
11H   ADHVDPTIPGTPFDSTPSIFDSQFFIETQLRGTLFPGTGGNPGEVESPLPGEIRLQSDHL  240

11WT  LARDSRTSCEWQSMVDNMPKIQNRFAATMLKMSLLGQNQADLIDCSDVIPTPPALVGKAH  300
11H   LARDPRTACEWQSMVDNMPKIQNRFAATMLKMSLLGQNVRDLIDCSDVIPTPPPLVGTAH  300

11WT  LPAGKVQSDVEQACATTPFPAIAADPGPVTAVPPVPPS   338
11H   LPAGKTQSDVEQACATTPFPTIPADPGPVTSVPPVPPS   338
```

Designed positions
Positions restricted for design
Mutations in these positions were excluded after visual inspection

Figure S2. Sequence alignment of 5H, 8H and 11H to their wildtype progenitor. PROSS-mutated positions are highlighted in green. Design in positions highlighted in gray were disallowed due to: proximity to one of the active sites or structural calcium ions, putative disulfide-bond forming cysteines or structural inconsistency in the best five models calculated by trRosetta. Mutations calculated by PROSS in positions highlighted in yellow were omitted after visual inspection due to one of the following reasons: low homologous-sequence data in the mutation region (PSSM with less than 10 sequences), formation or depletion of possible N-glycosylation site, radical mutation in the protein core (large hydrophobic to small hydrophobic, hydrophilic to hydrophobic substitutions, etc.), mutation in the heme's substrate pocket and mutation in possible contact with structural inconsistent regions.

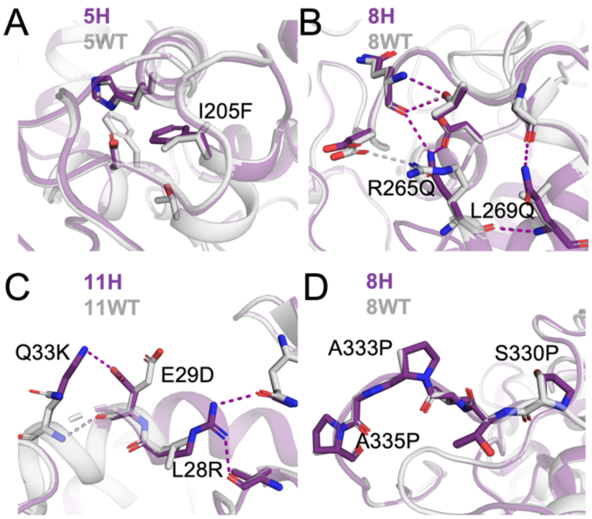

Figure S3. Examples of PROSS mutations in VP designs. (A) Ile205Phe in 5H improves core packing. (B) Arg265Gln and Leu269Gln in 8H and (C) Leu28Lys, Glu29Asp and Gln33Lys in 11H generate new hydrogen-bond networks. (D) Mutations at positions 330, 333 and 335 to Pro in 8H improve loop rigidity. In all panels, the PROSS-design model (purple) is superimposed onto the trRosetta-generated wildtype model (gray). Significant mutations, residues in their vicinity and the hydrogen bonds they form are presented in purple and gray sticks for the wildtype and designed models, respectively.

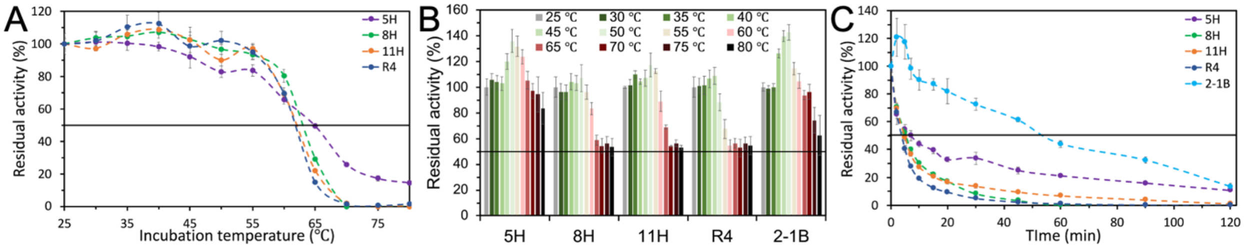

Figure S4. Thermal stabilities of selected VP designs. VPs (5H, 8H, 11H, VPL-R4 and 2-1B) were incubated for (A) 15 or (B) 10 minutes at temperatures ranging from 30 to 80 °C (in gradient steps of 5 °C), and their residual activity compared to the activity at 25 °C was measured (data not shown for 2-1B in A). (C) kinetic thermostability ($t_{1/2}$) profiles were determined by incubation of VP supernatants at 65 °C and measuring their residual activity at times 0-120 minutes, compared to the activity at time zero. All the results are the means ± S.D. from three independent experiments.

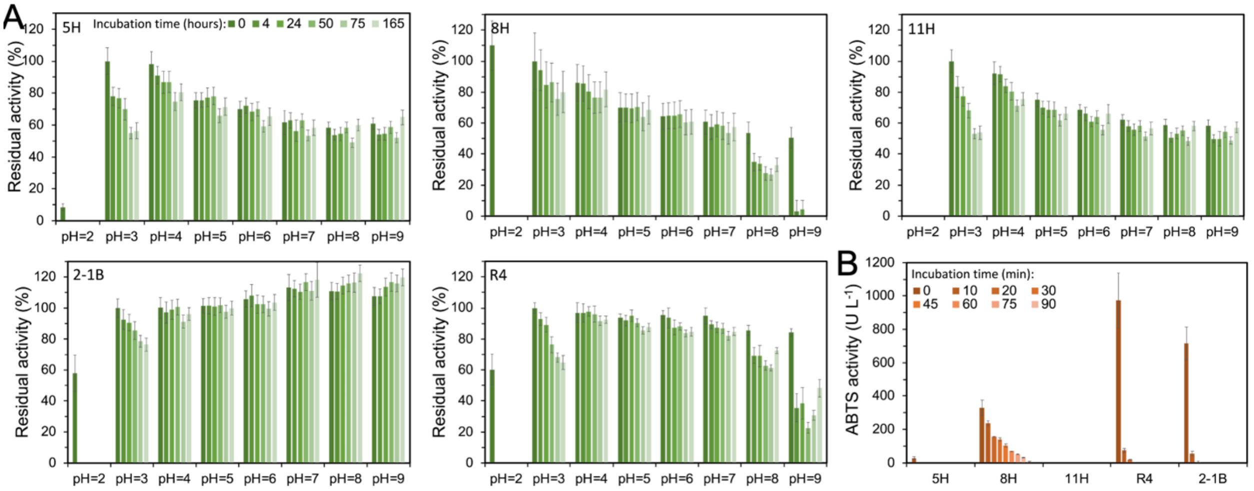

Figure S5. pH stabilities of selected VP designs. (A) VPs (5H, 8H, 11H, and VPL-R4 and 2-1B) were incubated at pH ranging from 2-9 using 100 mM citrate-phosphate-borate buffer, and their residual activity at times 0-165 hours, compared to the activity at pH=3 at time zero, was measured. (B) VPs were incubated at 100 mM citrate-phosphate-borate buffer pH=2, and their activity at times 0-150 minutes was measured. All the results are the means ± S.D. from three independent experiments.

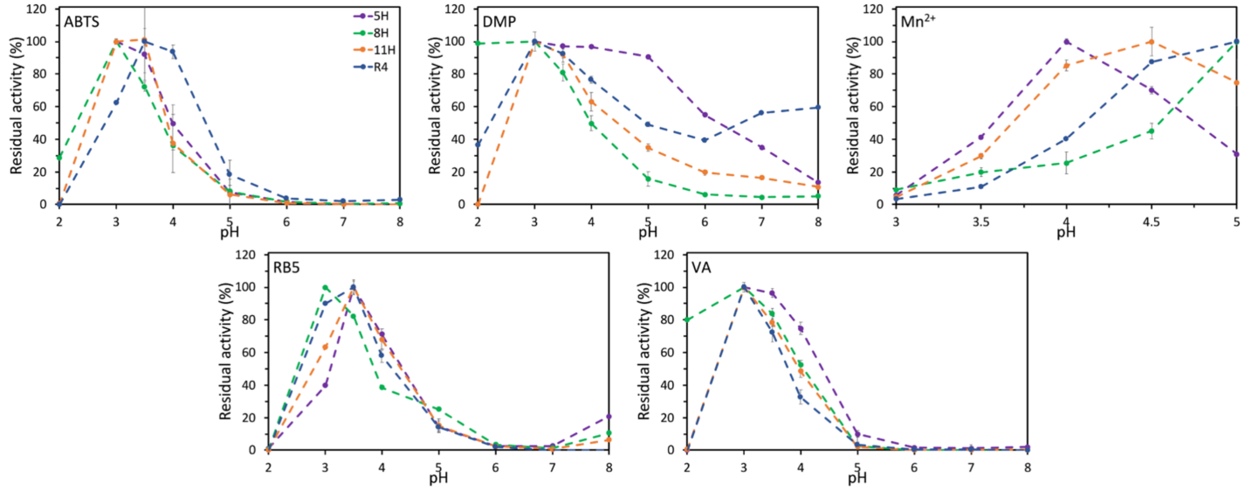

Figure S6. pH activity profiles of selected VP designs with versatile substrates. Purified 5H, 8H, 11H and R4 assayed for activity at range of pHs (pHs 2-9 to all substrates but Mn^{2+}, in which was tested at pH range of 3-5): ABTS, DMP, Mn^{2+}, VA and RB5. VPs activity was normalized to the activity at optimal pH for each protein-substrate pair. All the results are the means ± S.D. from three independent experiments.

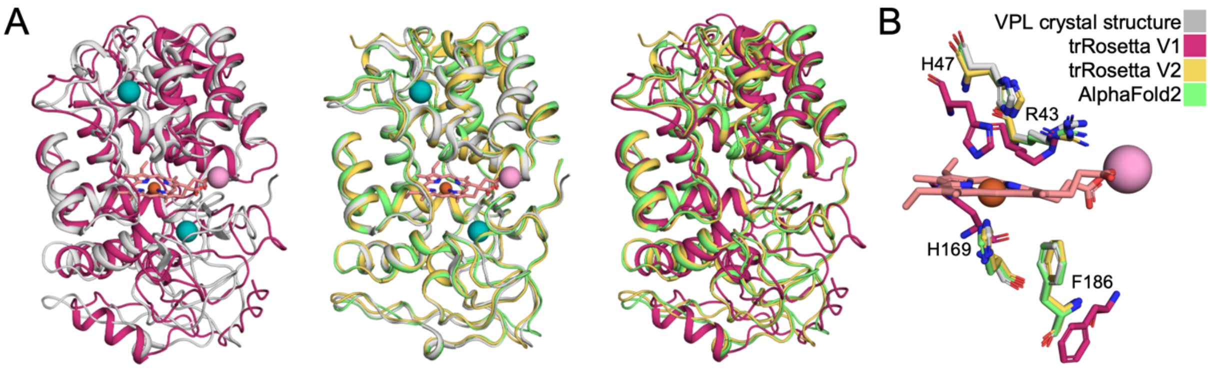

Figure S7. 2020's evolution of deep-learning based *ab initio* structure prediction methods. (A) Most reliable (top-ranked) models of a representative VP (VP11), superimposed onto one another and onto wildtype VPL crystal structure (PDB entry 3FJW; gray): trRosetta V1[9] (released in January 2020), trRosetta V2[10] (DeepAccNet-Rosetta, which became available via the Robetta server in December 2020) and AlphaFold2[13] (made available through Google Colab notebook in July 2021; green). VPL calcium and manganese ions are presented in blue and pink spheres, respectively, and the heme group in light pink sticks. (B) Close-up look onto the active site packing of wildtype VPL crystal structure and VP11 trRosetta V1, trRosetta V2 and AlphaFold2 models. VPL manganese ions and its heme group are presented as in (A). Position identities and numbers are relative to PDB entry 3FJW. Notice the close correspondence between trRosetta V2, AlphaFold2 and the VPL crystal structure in the core of the enzyme domain and within the heme binding pocket by contrast to the divergence of the trRosetta V1 model.

Supplementary References

(1) Grigoriev, I. V.; Nikitin, R.; Haridas, S.; Kuo, A.; Ohm, R.; Otillar, R.; Riley, R.; Salamov, A.; Zhao, X.; Korzeniewski, F.; Smirnova, T.; Nordberg, H.; Dubchak, I.; Shabalov, I. MycoCosm Portal: Gearing up for 1000 Fungal Genomes. *Nucleic Acids Res.* **2014**, *42* (Database issue), D699–D704.

(2) Savelli, B.; Li, Q.; Webber, M.; Jemmat, A. M.; Robitaille, A.; Zamocky, M.; Mathé, C.; Dunand, C. RedoxiBase: A Database for ROS Homeostasis Regulated Proteins. *Redox Biol* **2019**, *26*, 101247.

(3) Choi, J.; Détry, N.; Kim, K.-T.; Asiegbu, F. O.; Valkonen, J. P. T.; Lee, Y.-H. fPoxDB: Fungal Peroxidase Database for Comparative Genomics. *BMC Microbiol.* **2014**, *14*, 117.

(4) Almagro Armenteros, J. J.; Tsirigos, K. D.; Sønderby, C. K.; Petersen, T. N.; Winther, O.; Brunak, S.; von Heijne, G.; Nielsen, H. SignalP 5.0 Improves Signal Peptide Predictions Using Deep Neural Networks. *Nat. Biotechnol.* **2019**, *37* (4), 420–423.

(5) Edgar, R. C. MUSCLE: Multiple Sequence Alignment with High Accuracy and High Throughput. *Nucleic Acids Res.* **2004**, *32* (5), 1792–1797.

(6) Kumar, S.; Stecher, G.; Li, M.; Knyaz, C.; Tamura, K. MEGA X: Molecular Evolutionary Genetics Analysis across Computing Platforms. *Mol. Biol. Evol.* **2018**, *35* (6), 1547–1549.

(7) Stecher, G.; Tamura, K.; Kumar, S. Molecular Evolutionary Genetics Analysis (MEGA) for macOS. *Mol. Biol. Evol.* **2020**, *37* (4), 1237–1239.

(8) Madeira, F.; Park, Y. M.; Lee, J.; Buso, N.; Gur, T.; Madhusoodanan, N.; Basutkar, P.; Tivey, A. R. N.; Potter, S. C.; Finn, R. D.; Lopez, R. The EMBL-EBI Search and Sequence Analysis Tools APIs in 2019. *Nucleic Acids Res.* **2019**, *47* (W1), W636–W641.

(9) Yang, J.; Anishchenko, I.; Park, H.; Peng, Z.; Ovchinnikov, S.; Baker, D. Improved Protein Structure Prediction Using Predicted Interresidue Orientations. *Proc. Natl. Acad. Sci. U. S. A.* **2020**, *117* (3), 1496–1503.

(10) Hiranuma, N.; Park, H.; Baek, M.; Anishchenko, I.; Dauparas, J.; Baker, D. Improved Protein Structure Refinement Guided by Deep Learning Based Accuracy Estimation. *Nat. Commun.* **2021**, *12* (1), 1340.

(11) Goldenzweig, A.; Goldsmith, M.; Hill, S. E.; Gertman, O.; Laurino, P.; Ashani, Y.; Dym, O.; Unger, T.; Albeck, S.; Prilusky, J.; Lieberman, R. L.; Aharoni, A.; Silman, I.; Sussman, J. L.; Tawfik, D. S.; Fleishman, S. J. Automated Structure- and Sequence-Based Design of Proteins for High Bacterial Expression and Stability. *Mol. Cell* **2016**, *63* (2), 337–346.

(12) Weinstein, J. J.; Goldenzweig, A.; Hoch, S.-Y.; Fleishman, S. J. PROSS 2: A New Server for the Design of Stable and Highly Expressed Protein Variants. *Bioinformatics* **2020**. https://doi.org/10.1093/bioinformatics/btaa1071.

(13) Jumper, J.; Evans, R.; Pritzel, A.; Green, T.; Figurnov, M.; Ronneberger, O.; Tunyasuvunakool, K.; Bates, R.; Žídek, A.; Potapenko, A.; Bridgland, A.; Meyer, C.; Kohl, S. A. A.; Ballard, A. J.; Cowie, A.; Romera-Paredes, B.; Nikolov, S.; Jain, R.; Adler, J.; Back, T.; Petersen, S.; Reiman, D.; Clancy, E.; Zielinski, M.; Steinegger, M.; Pacholska, M.; Berghammer, T.; Bodenstein, S.; Silver, D.; Vinyals, O.; Senior, A. W.; Kavukcuoglu, K.; Kohli, P.; Hassabis, D. Highly Accurate Protein Structure Prediction with AlphaFold. *Nature* **2021**, *596* (7873), 583–589.

(14) Alcalde, M. Mutagenesis Protocols in Saccharomyces Cerevisiae by In Vivo Overlap Extension. In *In Vitro Mutagenesis Protocols: Third Edition*; Braman, J., Ed.; Humana Press: Totowa, NJ, 2010; pp 3–14.

(15) Viña-Gonzalez, J.; Gonzalez-Perez, D.; Ferreira, P.; Martinez, A. T.; Alcalde, M. Focused Directed Evolution of Aryl-Alcohol Oxidase in Saccharomyces Cerevisiae by Using Chimeric Signal Peptides. *Appl. Environ. Microbiol.* **2015**, *81* (18), 6451–6462.

(16) Garcia-Ruiz, E.; Gonzalez-Perez, D.; Ruiz-Dueñas, F. J.; Martínez, A. T.; Alcalde, M. Directed Evolution of a Temperature-, Peroxide- and Alkaline pH-Tolerant Versatile Peroxidase. *Biochem. J* **2012**, *441* (1), 487–498.
